# Prot2HG: a database of protein domains mapped to the human genome

**DOI:** 10.1093/database/baz161

**Published:** 2020-04-15

**Authors:** David Stanek, Dana M Bis-Brewer, Cima Saghira, Matt C Danzi, Pavel Seeman, Petra Lassuthova, Stephan Zuchner

**Affiliations:** 1 DNA Laboratory, Department of Paediatric Neurology, 2nd Faculty of Medicine, Charles University in Prague and University Hospital Motol, Prague, V Úvalu 84, 150 06 Czech Republic; 2 Department of Human Genetics and John P. Hussman Institute for Human Genomics, Miller School of Medicine, University of Miami, Miami, FL 33136, USA

## Abstract

Genetic variation occurring within conserved functional protein domains warrants special attention when examining DNA variation in the context of disease causation. Here we introduce a resource, freely available at www.prot2hg.com, that addresses the question of whether a particular variant falls onto an annotated protein domain and directly translates chromosomal coordinates onto protein residues. The tool can perform a multiple-site query in a simple way, and the whole dataset is available for download as well as incorporated into our own accessible pipeline. To create this resource, National Center for Biotechnology Information protein data were retrieved using the Entrez Programming Utilities. After processing all human protein domains, residue positions were reverse translated and mapped to the reference genome hg19 and stored in a MySQL database. In total, 760 487 protein domains from 42 371 protein models were mapped to hg19 coordinates and made publicly available for search or download (www.prot2hg.com). In addition, this annotation was implemented into the genomics research platform GENESIS in order to query nearly 8000 exomes and genomes of families with rare Mendelian disorders (tgp-foundation.org). When applied to patient genetic data, we found that rare (<1%) variants in the Genome Aggregation Database were significantly more annotated onto a protein domain in comparison to common (>1%) variants. Similarly, variants described as pathogenic or likely pathogenic in ClinVar were more likely to be annotated onto a domain. In addition, we tested a dataset consisting of 60 causal variants in a cohort of patients with epileptic encephalopathy and found that 71% of them (43 variants) were propagated onto protein domains. In summary, we developed a resource that annotates variants in the coding part of the genome onto conserved protein domains in order to increase variant prioritization efficiency.

**Database URL:** www.prot2hg.com

## Introduction

The widespread application of whole-exome sequencing (WES) and whole-genome sequencing (WGS) requires interpretation of individual sequence variants. Recommendations exist for variants in genes already associated with a disease (1). However, when searching for novel gene-to-disease associations or novel disease mechanisms, advanced annotation and interpretation is needed (2). Complementary methods to prioritize variants based on function or evolutionary properties such as sequence conservation, genetic effects and regulatory element annotations can serve to improve power and ultimately the success of disease studies (3).

To look for sequences conserved across species is an efficient strategy, and many tools were developed to search for evolutionarily conserved elements (4). The underlying assumption is that evolutionarily conserved regions tend to be less tolerant to mutations (5). Moreover, in the coding region of the genome, a domain-specific score system based on sequence homology is widely used for variants evaluation—SIFT (5), PolyPhen2 (6) or CADD (3, 7).

Protein domains are conserved structural entities, and information about a protein domain may provide clues as to the structure and/or function of the protein studied. Furthermore, it may help to establish evolutionary relationships across protein families (8). Last, but not least, it is useful in the interpretation of mutation studies.

To be able to determine whether specific human genetic variants fall onto annotated conserved protein domains might be helpful for studies of variant mapping in rare diseases, the global evaluation of genomic variation in relationship to protein architecture and the comprehensive evaluation of the population-level proteome as derived from genetically diverse human populations.

Several tools for next-generation sequencing variant annotation, which determine whether specific human genetic variants fall onto annotated conserved protein domains, have recently been published. For example, Protein Data Bank’s Map Genomic Position to Protein Sequence and 3D Structure (https://www.rcsb.org/pdb/chromosome.do) (9) provides users with multiple views and features. However, this tool is intended solely for a single query (one variant at a time), and to get the information about the protein domain, the user has to take additional steps via the UniProt hyperlink. Furthermore, download and incorporation into own pipeline are not possible.

Additionally, for example, in Ensembl’s Variant Effect Predictor (VEP) (http://grch37.ensembl.org/Homo_sapiens/Tools/VEP) (10), the user can annotate a batch of variants with ‘protein domains’. But to use this tool, the user has to upload the vcf file onto the Ensembl server, which often is not possible, due to security or data privacy reasons. Downloading from the Ensembl VEP is possible, however, and to get the desired information, the user only needs to use multiple tables.

Here we present a tool that can perform multiple queries (batch of 100 variants) in a simple way and give a quick, easily readable answer. Moreover, it is possible to download the entire dataset and integrate this annotation into a local pipeline.

The National Center for Biotechnology Information (NCBI) provides a comprehensive source of the human reference genome assembly (https://www.ncbi.nlm.nih.gov/refseq/) and is an essential resource for genomic, genetic and proteomic research (11). For proteins annotated on NM_transcript accessions, the information includes the known domain of proteins and their sequences, architecture and cross-species conservation. Though the NCBI resource includes this information, it is only at the protein level. Therefore, in Reference Sequence (RefSeq), there is no connection to genomic coordinates, and its usefulness for variant annotation is limited.

We developed a resource that annotates variants in the coding part of the genome onto conserved protein domains in an easy-to-use way. This could help to make variant interpretation more efficient. The resource thus provides this missing connection for large-scale genome/proteome studies and surpasses prioritization of variants. Our data are freely accessible through a dedicated open web-based query tool (www.prot2hg.com) or as a downloadable dataset. Moreover, data were incorporated into the GENESIS platform for analysis and matchmaking of exome and genome data from rare diseases (tgp-foundation.org).

## Materials and methods

**Figure 1:**
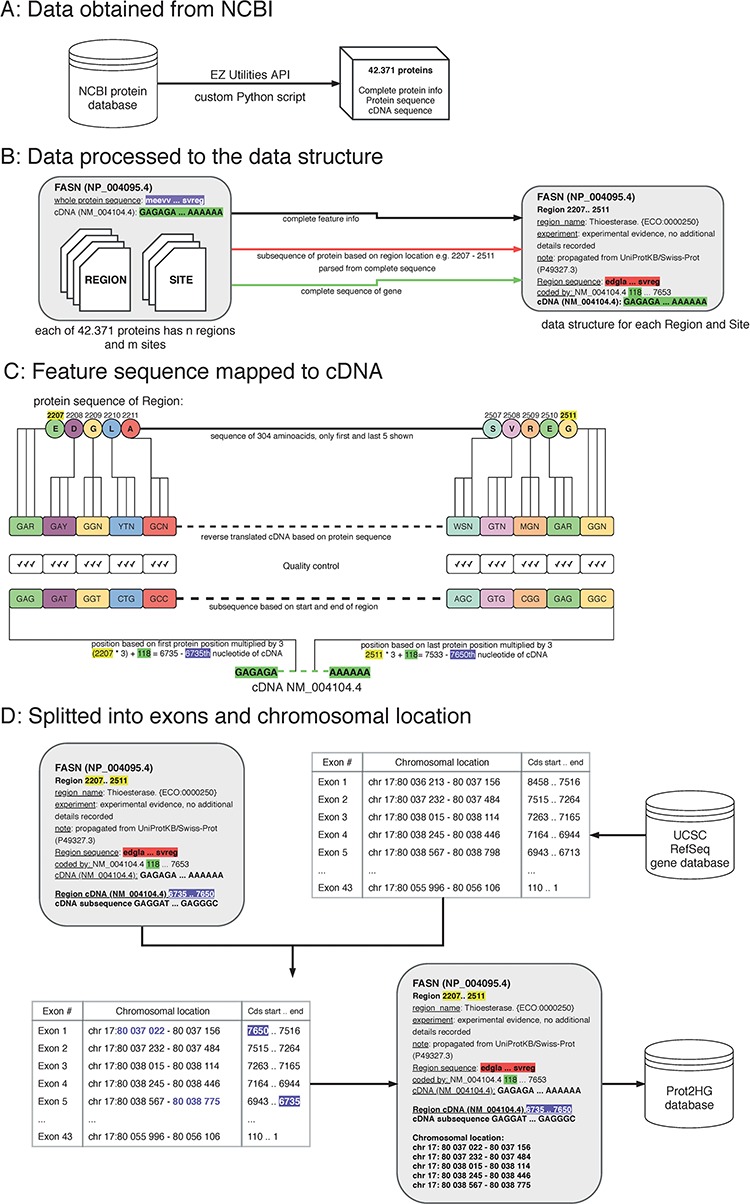
The process of protein domain mapping to human genome. (A) Data obtained from NCBI—data were downloaded about all proteins and stored into data structure for further analysis. (B) Data processed to the data structure—during this step, we record each protein domain with its subsequence and complete information. (C) Feature sequence mapped to cDNA—in this step, the protein subsequence is translated into a DNA sequence. A matching sequence in cDNA is then found and compared to a translated sequence to give a match. (D) Data split into exons and chromosomal location was assigned—some domains cover more than one exon, and in this step, using the UCSC gene database, we identify the chromosome location of every exon that codes to the domain. Then, the completed record is stored in the Prot2HG database.

A protein domain is a segment within a protein with a known sequence and a previously described function. To ultimately map each protein domain to a chromosomal location, a workflow with four key steps was implemented: obtain data from NCBI; process data to create a custom data structure; map protein domain locations to cDNA sequence; and map cDNA sequence location to hg19 genomic location (Figure 1).

### Obtain data from NCBI

The records published in the NCBI/RefSeq protein database are presented as sets of feature tables providing structured information about protein sequence and length and all known domains. Each domain also has a feature table where additional information is stored (type of domain, length, source of the observation and nucleotide position). In this analysis, two types of protein domains were used: sites and regions.

### Definitions

A protein site is a protein domain with a shorter sequence and, often, specifically known functions. Many protein sites reside within larger protein domains. Examples of such protein sites are transmembrane regions and nitrosylation or phosphorylation sites.

A protein region is part of a protein with a known function, usually with a length of over 100 amino acids. It is a part of a protein that can adopt a particular 3D structure (12). Typical protein regions include zinc fingers, WD40 repeats and leucine-rich repeats.

The cDNA and protein sequences, feature tables and RefSeq Gene identification from the NCBI protein database for each protein were downloaded via the EZ Utilities application programming interface (API) using custom Python scripts (13).

### Process data to create a custom data structure

Custom Python scripts were used to transform the NCBI data structure. Data were split into blocks of structured text for each protein followed by extraction of the amino acid sequence, cDNA sequence, RefSeq identifier (NM_ID) and coding sequence information to determine sequence shift. The shift (stored in the ‘coded_by’ line in Figure 1B, highlighted in green) is crucial for matching the translated sequences to correct the cDNA. Each protein region and site contained the following storage information: domain name, experiment description and note, start/end locations in amino acid sequence (used to parse protein subsequences) and cDNA sequence.

### Map protein domain sequences to cDNAs

A double-verification system was implemented to ensure that amino acid sequences were properly mapped to the cDNA sequence. The input for this step is a custom data structure (from the previous step), with protein subsequence, complete sequence of a gene and the location of the start and end of the protein domain. To begin mapping, each protein subsequence (amino acid) was converted into its nucleic acid sequence. Due to codon degeneracy, with 64 possible triplets coding for only 20 different amino acids, most amino acids are inserted into a growing polypeptide chain in response to two or more different triplets in the mRNA (14). To resolve this, an International Union of Pure and Applied Chemistry (IUPAC) notation was used to avoid multiplicity (15). For example, glycine results from four codons (GGT, GGC, GGA and GGG) and the IUPAC representation is GGN. The reverse-translated sequences were mapped to the RefSeq cDNA sequence by multiplying the protein sequence location (Figure 1, highlighted in yellow) by three and adding the shift (Figure 1, highlighted in purple). As a final quality control, reverse-translated sequences were compared with the gene subsequences and mapping scores were computed. Each mapping score is a ratio between the number of matching nucleotides and all nucleotides in the domain (value between 0.0 and 1.0). The information about gene subsequence, such as starting positions, end positions and nucleotide sequences, was then added to our custom data structure.

### Split into exons and chromosomal location

All chromosomal intervals for each exon from the University of California Santa Cruz (UCSC) RefSeq gene database were downloaded via the MySQL API (https://genome.ucsc.edu/) to annotate each protein with a chromosomal location. The exon table containing the number of exons, exon chromosomal location and coding strand location for each NM_ID was retrieved. The UCSC exon table information was combined with our computed cDNA start/end locations to determine which exons are translated into domains. The location of each exon within the domain interval was stored. Lastly, the Prot2HG database (MySQL) was created to store the annotated data with web-based query tools, enabling the user to find an annotation for individual variants.

## Results

### Statistics of Prot2HG

We analyzed a total of 42 371 feature tables for proteins and matching genes. These proteins contain 808 886 protein domains, of which 190 760 were regions and 616 126 were protein sites. Protein regions are longer elements within coding genes (mean length 315.6 bp). Sites were shorter with a mean length of 7.3 bp (online supplementary material Table 2).

For each record, the following details are provided: gene name; RefSeq ID—protein (NP_) and gene (NM_); mapped strand (+/−); type of domain (site/region); domain name; reverse mapping score (0.00–1.00); chromosomal location; and the feature table note with further information. The number of records in the database is significantly higher than the number of protein domains due to splitting into exons.

**Figure 2:**
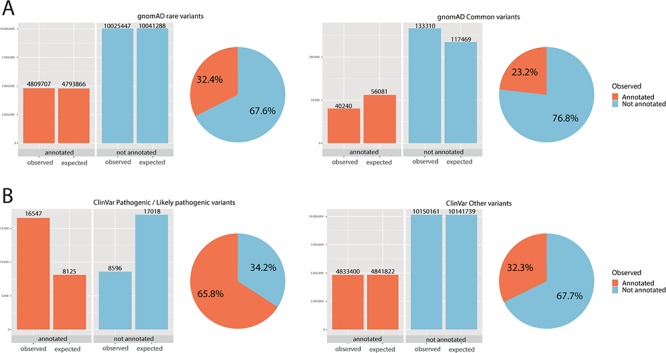
Chi-square statistics. From observed (real) data, we were able to calculate the expected data distribution. The difference between the two columns (observed vs. expected) is proportional to the dependence of the data and accordingly statistical significance. The difference is also proportional to the odds ratio. The pie chart is calculated from observed (real) data only. (A) For rare variants, 32% were annotated, while only 23% of common variants fall onto a conserved protein domain. The results show the dependence of distribution, and the difference was evaluated as significant with *P* < 0.01 and odds ratio of 1.59. (B) For ClinVar, the difference was even more obvious: 66% of pathogenic/likely pathogenic variants were annotated, while only 32% of other ClinVar variants fall onto a domain. The results show the dependence of distribution, and the difference was evaluated as significant with *P* < 0.01 and odds ratio of 4.04.

### Data access

The entire database is accessible at www.prot2hg.com. The website is built on a PHP server using the Twitter Bootstrap HTML template and connected to the MySQL database. In the home tab (online supplementary material Figure 1), a textbox allows for variant and chromosomal location input (up to 100 variants). After submitting variants of interest, the query results are displayed in a table. Users can browse the data and export annotated variants into a CSV file.

The database download tab enables users to download the database for their own annotation and incorporation into their own pipeline and is free to use. The data are available in JSON, SQL and CSV formats, and the schema of the table is shown in online supplementary material Table 1.

### Annotation analysis and statistics of distribution

Variants in the Genome Aggregation Database (gnomAD) exome database (16), in addition to pathogenic/likely pathogenic variants in the ClinVar database (17), were used as test data and annotated by the Prot2HG database. Each variant was evaluated on the basis of population frequency (rare: <1%; common ≥1%), ClinVar status (pathogenic/likely pathogenic) and propagation onto a protein domain (region or site). Results for each chromosome are listed in online supplementary material Table 2.

Statistical evaluation with a chi-square test was done to test the random distribution of the data (Figure 2). The independence between rare variants in the group of annotated and not annotated variants was queried. Moreover, the relationship between the number of pathogenic/likely pathogenic variants in the group of annotated and not annotated variants was tested.

Significantly more rare than common variants were annotated onto a domain (for rare variants 32%, for common variants 23%, *P* < 0.01, odds ratio 1.59). Further, for ClinVar pathogenic/likely pathogenic, 66% of variants fall on to an annotated domain, while only 32% of variants without pathogenic/likely pathogenic status were annotated. Again, these results showed a significant and dependent relationship between ClinVar pathogenic status and annotation onto a protein domain was observed (*P* < 0.01, odds ratio 4.04). Chi-square statistics are summarized in online supplementary material Table 3.

### Clinical example of usage

We tested the concept of the Prot2HG database with our own real variant dataset. This dataset consisted of 60 previously confirmed pathogenic or likely pathogenic variants in a cohort of patients with epileptic encephalopathy—a severe early-onset disorder with full penetrance and with Mendelian inheritance [online supplementary material Table 3 (18)].

Out of these 60 causal variants, 71% of them (43) were propagated onto protein domains. From the functional point of view, these were mostly ion transport regions (14x), transmembrane regions (6x) and ion-channel transmembrane regions (5x). Many of the variants were annotated onto multiple domains (43 variants onto 93 domains).

## Discussion

Genomics has enormously improved the diagnosis of human diseases. However, for whole-exome sequencing, the diagnostic yield varies between 20 and 50% (19). It is believed that whole-genome sequencing might be beneficial by addressing the limitations of a targeted approach. While the limitation of target/non-coding region is overcome by WGS, the clinical and functional interpretation of the variants remains a challenge (20). Thus, additional, novel ways of annotation and evaluation of variants are needed. Currently, population databases such as gnomAD and various *in silico* prediction tools are helpful. Moreover, phenotype driven analysis (21) is essential. Functional annotations are crucial, and information about the precise location of variation in conserved regions of protein products might be useful. Our work makes this annotation easily accessible.

The usefulness of this tool has been tested by analyzing a real dataset. Firstly, gnomAD variants were tested. GnomAD variants were divided into two groups (common variants ≥1%; rare variants <1%). We observed a significant difference between a proportion of variants that were annotated onto a protein domain (23% for common, 32% for rare variants), and a chi-square test showed this difference to be significant. As protein domains are conserved regions of the genome, these regions are thus less likely to be hypervariable, and this may explain the difference in the distribution.

Similarly, data from ClinVar were extracted. Variants designed as pathogenic/likely pathogenic in ClinVar were annotated onto a domain in 66%, while only 32% remaining variants (not pathogenic/likely pathogenic) were annotated onto a domain. The chi-square test was used for analysis; the difference was evaluated as significant and may support the hypothesis that pathogenic/likely pathogenic variants are more often distributed through known protein domains.

These results highlight the utility of such annotations. For variants in genes with known disease associations, this approach could help make variant analysis more efficient. Moreover, when searching for novel disease-to-gene associations, such prioritization of variants might be useful, because it provides clues as to the function of the protein. And for certain types of disorders (epileptic encephalopathy being a representative example), it is a fundamental piece of information to know that a variant is annotated onto an ion transport or transmembrane protein domain. We demonstrated the utility of Prot2HG and its impact on variant interpretation through retrospective analysis of clinical Next Generation Sequencing dataset consisting of 60 previously confirmed pathogenic or likely pathogenic variants in a cohort of patients with epileptic encephalopathy. It was found that most of the variants (71%) were truly propagated onto protein domains.

## Conclusion

Here we introduce an annotation with NCBI protein features that addresses the question of whether a particular variant falls onto an annotated (known) protein domain. The database is available at www.prot2hg.com. The tool will expand variant annotation tools available, especially for rare diseases. Functional analysis and interpretation of variants from NGS based on possible functional consequences is the future. We suggest this annotation is particularly useful when analyzing/re-analyzing data from WES while searching for novel gene-to-disease associations.

## Funding

This work was supported by the Charles University, project GA UK [grant number 388217] and supported by Ministry of Health of the Czech Republic [grant number 16-30206].

*Conflict of interest*. None declared.

## Supplementary Material

Supplementary_file_for_baz161
