# Supplementary material for: Prot2HG: a database of protein domains mapped to the human genome
Source: Database (Oxford). 2020 Apr 15;2020:baz161. doi: 10.1093/database/baz161 (PMC7157182; doi:10.1093/database/baz161)
Supplement: Supplementary_file_for_baz161 [file supplementary_file_for_baz161.pdf]

Supplementary file for:

Prot2HG: a database of protein domains mapped to the human genome

Stanek David[1], Bis-Brewer Dana M[2], Saghira Cima[2], Danzi Matt C[2], Lassuthova Petra[1*], Seeman Pavel[1] and Zuchner Stephan[2]

[1] Department of Paediatric Neurology, DNA Laboratory, 2nd Faculty of Medicine, Charles University in Prague and University Hospital Motol, Prague, Czech Republic;

[2] Department of Human Genetics and John P. Hussman Institute for Human Genomics, Miller School of Medicine, University of Miami, Miami, FL 33136, USA;

[*] **Corresponding author:**

Petra Lassuthova**,** Department of Paediatric Neurology, DNA Laboratory, 2nd Faculty of Medicine, Charles University in Prague and University Hospital Motol, Prague, Czech Republic; petra.lasssuthova@fnmotol.cz, phone: +420 224 436 789, fax: +420 224 435 820

Content:

Table 1: Database schema of prot2hg p.2

Figure 1: Overview of prot2hg homepage p.3

Table 2: Number of variants in the groups p.5

Table 3: Results of Chi-square test of independence p.6

Table 4: Testing data / 60 causal pathogenic mutations p.8

Table 1: Database schema of prot2hg

Id	identification number of each line
Gene	the name of gene listed in RefSeq
Protein_ID	RefSeq identifier of protein
Gene_ID	RefSeq identifier of gene which is translated into product with Protein_ID
Strand	the strand of the DNA, where gene is located [+ / -]
Type	type of protein domain [Region / Site]
Feature_name	name of the domain
Prot_start	starting posititon of domain in protein sequence
Prot_end	ending posititon of domain in protein sequence
Cds_start	starting posititon of domain in cDNA of gene
Cds_end	ending posititon of domain in cDNA of gene
Cds_length	the length of domain in bp
Rev_trans_map	score, describing the quality of reverse translation [0
Chr_Start	starting posititon of domain in chromosomal DNA
Chr_End	ending posititon of domain in chromosomal DNA
Note	the commentary about domain listed in NCBI
CDD	Conserved domain database identification
Chrom	chromosome, in “chr_” format

Figure 1: Overview of prot2hg homepage.

User enters variants into input textbox, after submitting the query are all annotated variants shown in table with export options and with available search

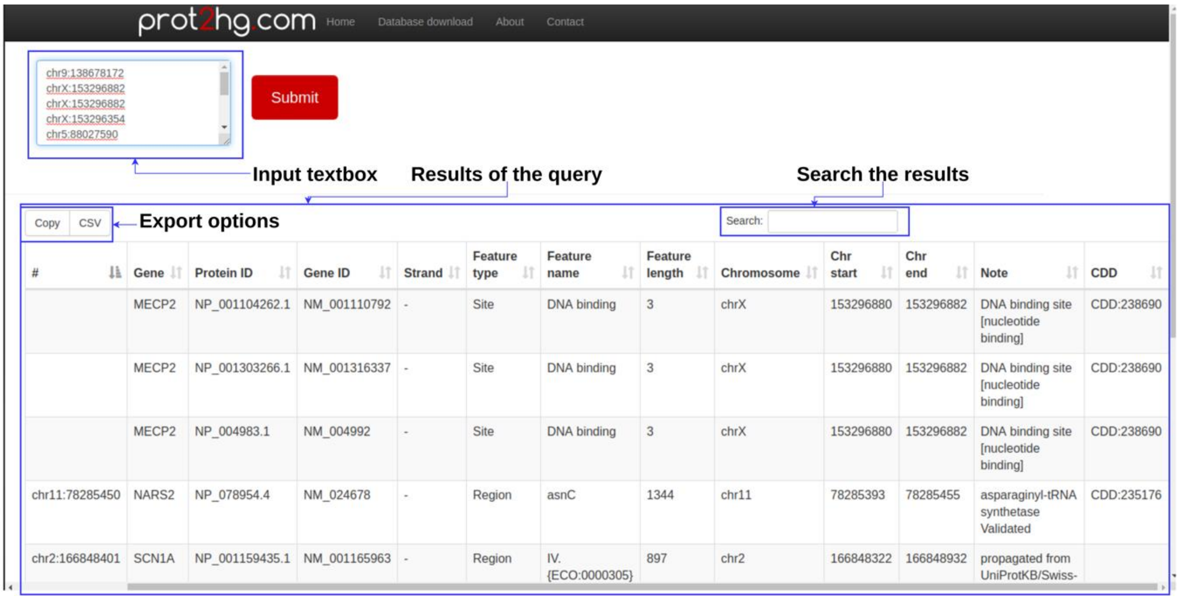

Text

Table 2: Number of variants in the groups

Chr	All	Regions	Sites	A_All	A_Common	A_Rare	A_ClinVar	N_All	N_Common	N_Rare	N_ClinVar
**chr1**	483643	474563	51896	483643	4238	479405	1431	1011316	13250	998066	734
**chr2**	341035	335633	32648	341035	2412	338623	1591	737593	8887	728706	646
**chr3**	285275	279878	32143	285275	1956	283319	957	555713	6840	548873	370
**chr4**	185295	181604	19817	185295	1528	183767	450	378354	5000	373354	297
**chr5**	224329	220643	23646	224329	1710	222619	688	425803	5331	420472	364
**chr6**	243931	239567	28150	243931	2881	241050	646	489075	7775	481300	364
**chr7**	218162	214360	26004	218162	1755	216407	800	519136	7264	511872	322
**chr8**	166582	163881	16546	166582	1186	165396	440	371435	4819	366616	404
**chr9**	196690	192983	19418	196690	1590	195100	703	425407	5400	420007	289
**chr10**	181682	179084	15341	181682	1519	180163	490	402627	5642	396985	258
**chr11**	303660	296116	45762	303660	2781	300879	1227	608238	7484	600754	635
**chr12**	256844	252106	28713	256844	2086	254758	770	525411	6574	518837	337
**chr13**	78567	77195	8053	78567	597	77970	663	174533	2168	172365	465
**chr14**	153645	150797	16482	153645	1236	152409	448	351843	4871	346972	195
**chr15**	162036	159527	14388	162036	1308	160728	609	371689	5081	366608	411
**chr16**	227293	222200	24486	227293	1813	225480	959	513548	6514	507034	473
**chr17**	298535	293384	33162	298535	2322	296213	1107	599201	7428	591773	861
**chr18**	67997	66518	7537	67997	558	67439	230	154690	2112	152578	114
**chr19**	379026	372784	49494	379026	3499	375527	873	682449	9668	672781	364
**chr20**	122527	120529	11936	122527	1023	121504	282	252663	3170	249493	124
**chr21**	51210	50616	3715	51210	513	50697	212	110728	1691	109037	95
**chr22**	109532	107599	10567	109532	968	108564	335	248318	3583	244735	166
**chrX**	112451	110554	11403	112451	761	111690	636	248987	2758	246229	308
**SUM**	**4849947**	**4762121**	**531307**	**4849947**	**40240**	**4809707**	**16547**	**10158757**	**133310**	**10025447**	**8596**

Legend:
Chr – chromosome; All – number of all annotated variants; Regions – number of variants annotated into Regions; Sites – number of variants annotated into Sites; A_Common – number of annotated polymorphism variants (with allele frequency in gnomAD > 0.01); A_Rare – number of annotated rare variants (with allele frequency in gnomAD < 0.01); A_ClinVar – number of annotated variants annotated as Pathogenic or Likely pathogenic in ClinVar;N_all – number of not annotated variants; N_Common – number of not-annotated polymorphism variants (with allele frequency in gnomAD > 0.01); A_Rare – number of not-annotated rare variants (with allele frequency in gnomAD < 0.01); A_ClinVar – number of not-annotated variants annotated as Pathogenic or Likely pathogenic in ClinVar;

Table 3: Results of Chi-square test of independence

Rare and common variants comparison

**Regions and Sites**	**Rare**	**Common**
Annotated	4 809 707	40 240
Not annotated	10 025 447	133 310
**An association was observed $\chi 2(1) = 6688.357$, $p < 0.01$.**		
**Odds ratio**	**1,59**	

**Regions**	**Rare**	**Common**
Annotated regions	4 722 630	39 491
Not annotated	10 025 447	133 310
**An association was observed $\chi 2(1) = 6607.487$, $p < 0.01$.**		
**Odds ratio**	**1,59**	

**Sites**	**Rare**	**Common**
Annotated sites	526 862	4 445
Not annotated	10 025 447	133 310
**An association was observed $\chi 2(1) = 898.024$, $p < 0.01$.**		
**Odds ratio**	**1,58**	

Legend:

Chi-square statistics with contingency table analysis was done. The two directions of classification (rows and columns) are dependent at $p<0.01$. Odds ratio 1.59.

Table 3: Results of Chi-square test of independence (continue)

Pathogenic variants and not pathogenic variants in ClinVar comparison

**Regions and Sites**	**Clinvar**	**NOT Clinvar**
Annotated	16 547	4 833 400
Not annotated	8 596	10 150 161
**An association was observed χ2(1) = 12920.354, p < 0.01.**		
**Odds ratio**	**4,04**	

**Regions**	**Clinvar**	**NOT Clinvar**
Annotated regions	16 334	4 745 787
Not annotated	8 596	10 150 161
**An association was observed χ2(1) = 12976.833, p < 0.01.**		
**Odds ratio**	**4,06**	

**Sites**	**Clinvar**	**NOT Clinvar**
Annotated sites	2 211	529 096
Not annotated	8 596	10 150 161
**An association was observed χ2(1) = 5494.876, p < 0.01.**		
**Odds ratio**	**4,93**	

Legend:

Chi-square statistics with contingency table analysis was done. The two directions of classification (rows and columns) are dependent at $p<0.01$. Odd ratio 4.04.

Table 4: Testing data / 60 causal pathogenic mutations and their annotation onto domains

Variant	Chr	Position	Protein ID	Annotated domain
**Ion transport protein:**				
HCN1_NM_021072.3 c.1189A>G p.Ile397Leu	chr5	45 396 635	NP_066550.2	ion transport protein
KCNQ2_NM_172107.2 c.701C>T p.Thr234Ile	chr20	62 073 874	NP_004509.2	ion transport protein
KCNQ2_NM_172107.2 c.826A>C p.Thr276Pro	chr20	62 071 052	NP_004509.2	ion transport protein
KCNQ2_NM_172107.2 c.913_915delTTC p.Phe305del	chr20	62 070 962	NP_004509.2	ion transport protein
SCN1A_AB093548.1:c.542A>G p.Glu181Gly	chr2	166 911 208	NP_001159435.1	ion transport protein
SCN1A_NM_001165963.1 c.5384A>G p.Glu1795Gly	chr2	166 848 401	NP_001159435.1	ion transport protein
SCN1A_NM_001165963.1c.1178G>A p.Arg393His	chr2	166 903 479	NP_001159435.1	ion transport protein
SCN1A_NM_001165963.1c.4384dup p.Tyr1462Leufs*24	chr2	166 854 639	NP_001159435.1	ion transport protein
SCN1A_NM_001202435.1c.1244T>A p.Ile415Lys	chr2	166 903 413	NP_001159435.1	ion transport protein
SCN2A_NM_001040142.1 c.2774T>C p.Met925Thr	chr2	166 201 276	NP_001035232.1	ion transport protein
SCN2A_NM_021007.2:c.4756C>T p.Arg1586Cys	chr2	166 243 460	NP_001035232.1	ion transport protein
SCN8A_NM_014191.3 c.2549G>A p.Arg850Gln	chr12	52 159 459	NP_001317189.1	ion transport protein
SCN8A_NM_014191.3 c.4921C>G p.Leu1641Val	chr12	52 200 191	NP_001317189.1	ion transport protein
SCN8A_NM_014191.3c.4850G>T p.Arg1617Leu	chr12	52 200 120	NP_001317189.1	ion transport protein
**Transmembrane region:**				
GABRB3_NM_000814.5 c.841A>G p.Thr281Ala	chr15	26 806 318	NP_000805.1	transmembrane region
GABRB3_NM_000814.5:c.863C>A p.Thr288Asn	chr15	26 806 296	NP_000805.1	transmembrane region
GRIN1_NM_007327.3c.1643G>A p.Arg548Gln	chr9	140 058 120	NP_000823.4	transmembrane region
KCNQ2_NM_172107.2 c.701C>T p.Thr234Ile	chr20	62 073 874	NP_742105.1	transmembrane region
KCNQ2_NM_172107.2 c.913_915delTTC p.Phe305del	chr20	62 070 962	NP_742105.1	transmembrane region
SLC13A5_NM_177550.3c.425C>T p.Thr142Met	chr17	6 607 319	NP_808218.1	transmembrane region
**Ion channel:**				
GABRB3_NM_000814.5 c.841A>G p.Thr281Ala	chr15	26 806 318	NP_000805.1	ion-channel transmembrane region
GABRB3_NM_000814.5:c.863C>A p.Thr288Asn	chr15	26 806 296	NP_000805.1	ion-channel transmembrane region
GABRG2_NM_000816.3 c.968G>A p.Arg323Gln	chr5	161 576 159	NP_000807.2	ion-channel transmembrane region
KCNQ2_NM_172107.2 c.826A>C p.Thr276Pro	chr20	62 071 052	NP_004509.2	ion-channel transmembrane region
KCNQ2_NM_172107.2 c.913_915delTTC p.Phe305del	chr20	62 070 962	NP_004509.2	ion-channel transmembrane region
**Cation transporter:**				
GABRB3_NM_000814.5 c.841A>G p.Thr281Ala	chr15	26 806 318	NP_000805.1	cation transporter family protein
GABRB3_NM_000814.5:c.863C>A p.Thr288Asn	chr15	26 806 296	NP_000805.1	cation transporter family protein
GABRG2_NM_000816.3 c.968G>A p.Arg323Gln	chr5	161 576 159	NP_000807.2	cation transporter family protein
**Others:**				
ALDH7A1_NM_001182.4c.518-14_518delinsCA	chr5	125 912 902	NP_001173.2	NAD+-dependent dehydrogenase
ALDH7A1_NM_001182.4c.518-14_518delinsCA	chr5	125 912 902	NP_001173.2	nitrosylation - polypeptide binding
FOLR1_NM_000802.3:c.439C>T p.Arg147Cys	chr11	71 906 737	NP_000793.1	folate receptor
GRIN1_NM_007327.3c.1643G>A p.Arg548Gln	chr9	140 058 120	NP_000823.4	ligand-gated ion channel
GRIN1_NM_007327.3c.2443G>A p.Gly815Arg	chr9	140 056 634	NP_000823.4	ligand-binding domain
GRIN2A_NM_000833.4:c.593G>A p.Trp198*	chr16	10 032 230	NP_000824.1	LIVBP like domain
HUWE1:c.12195 G>C p.Trp4065Cys	chrX	53 563 571	NP_113584.3	HECT domain
HUWE1:c.12195 G>C p.Trp4065Cys	chrX	53 563 571	NP_113584.3	HECT domain
IQSEC2NM 001111125.2 c.3206G>C p.Arg1069Pro	chrX	53 267 398	NP_001104595.1	PH domain
KCNJ10_NM_002241.4:c.313C>T p.His105Tyr	chr1	160 012 010	NP_002232.2	potassium channel - inward rectifier
MECP2_NM_001110792.1:c.433C>T p.Arg145Cys	chrX	153 296 882	NP_001104262.1	Methyl-CpG binding domain
MECP2_NM_001110792.1:c.433C>T p.Arg145Cys	chrX	153 296 882	NP_001104262.1	DNA binding
MECP2_NM_004992.3 c.925C>T p.Arg309Trp	chrX	153 296 354	NP_004983.1	NCOR2 interaction
MEF2C_NM_002397.4 c.766C>T p.Arg256*	chr5	88 027 590	NP_001180277.1	MADS box-containing tr.factors
NARS2:c.83_84del p.Leu28Glnfs*17	chr11	78 285 450	NP_078954.4	asparaginyl-tRNA synthetase
PCDH19_NM_001184880.1c.698A>G p.Asp233Gly	chrX	99 662 898	NP_001098713.1	tandem repeat domain
PCDH19_NM_001184880.1c.698A>Gp.Asp233Gly	chrX	99 662 898	NP_001098713.1	nitrosylation - ion binding
PPP2R5D:c.1267_1270delCTCT p.Leu423fs	chr6	42 977 075	NP_001257405.1	protein phosphatase
PURA_NM_005859.4 c.812_814del p.Phe271del	chr5	139 494 575	NP_005850.1	PurA ssDNA and RNA-binding prote
PURA_NM_005859.4 c.812_814del p.Phe271del	chr5	139 494 575	NP_005850.1	DNA/RNA binding repeat
SLC13A5_NM_177550.3c.425C>T p.Thr142Met	chr17	6 607 319	NP_001137310.1	Di- and tricarboxylate transporter
SLC13A5_NM_177550.3c.425C>T p.Thr142Met	chr17	6 607 319	NP_001137310.1	Anion permease ArsB/NhaD.
SLC25A22_NM_001191060.1:c.347G>A p.Cys116Tyr	chr11	792 935	NP_001177989.1	mitochondrial carrier protein
SLC25A22_NM_001191060.1:c.347G>A p.Cys116Tyr	chr11	792 935	NP_001177989.1	Solcar2
SLC25A22_NM_001191060.1:c.347G>A p.Cys116Tyr	chr11	792 935	NP_001177989.1	ADP/ATP transporter
SLC25A22_NM_001191060.1:c.524G>T p.Arg175Leu	chr11	792 616	NP_001177989.1	mitochondrial carrier protein
SLC25A22_NM_001191060.1:c.524G>T p.Arg175Leu	chr11	792 616	NP_001177989.1	Solcar2
SLC25A22_NM_001191060.1:c.524G>T p.Arg175Leu	chr11	792 616	NP_001177989.1	ADP/ATP transporter
STXBP1_NM_003165.3c.1654T>C p.Cys552Arg	chr9	130 444 791	NP_001027392.1	Sec1 family
TREX1_NM_016381.3c.10621072del p.Leu354Phefs*22	chr3	48 508 951	NP_057465.1	ER localization
TREX1_NM_016381.3c.1072A>C p.Thr358Pro	chr3	48 508 961	NP_057465.1	ER localization
UBTF:c.628 G>A p.Glu210Lys	chr17	42 290 219	NP_055048.1	HMGB-UBF_HMG-box
WDR45_NM_007075.3 c.511C>T p.Gln171*	chrX	48 933 533	NP_001025067.1	WD40 repeat domain
WDR45_NM_007075.3 c.654del p.Arg219Alafs*69	chrX	48 933 277	NP_001025067.1	WD40 repeat domain
WDR45_NM_007075.3 c.970_971del p.Val324Hisfs*17	chrX	48 932 799	NP_001025067.1	WD7 repeat domain
